# Determination of VEGFR-2 (KDR) 604A>G Polymorphism in Pancreatic Disorders

**DOI:** 10.3390/ijms18020439

**Published:** 2017-02-17

**Authors:** Vlad Pădureanu, Mihail Virgil Boldeanu, Ioana Streaţă, Mihai Gabriel Cucu, Isabela Siloşi, Lidia Boldeanu, Maria Bogdan, Anca Ştefania Enescu, Maria Forţofoiu, Aurelia Enescu, Elena Mădălina Dumitrescu, Dragoş Alexandru, Valeriu Marian Şurlin, Mircea Cătălin Forţofoiu, Ileana Octavia Petrescu, Florin Petrescu, Mihai Ioana, Marius Eugen Ciurea, Adrian Săftoiu

**Affiliations:** 1 Department of Internal Medicine, University of Medicine and Pharmacy of Craiova, 2 Petru Rares Street, 200349 Craiova, Romania; vldpadureanu@yahoo.com (V.P.); catalin1972@hotmail.com (M.C.F.); petrescu.florin@yahoo.com (F.P.); 2 Department of Immunology, University of Medicine and Pharmacy of Craiova, 2 Petru Rares Street, 200349 Craiova, Romania; isabela_silosi@yahoo.com; 3 Medico Science SRL—Stem Cell Bank Unit, 1B Brazda lui Novac Street, 200690 Craiova, Romania; barulidia@yahoo.com; 4 Human Genomics Laboratory, University of Medicine and Pharmacy of Craiova, 2 Petru Rares Street, 200349 Craiova, Romania; ioana.streata@yahoo.com (I.S.); cucu_mihai_gabriel@yahoo.com (M.G.C.); mihaiioana_romania@yahoo.com (M.I.); 5 Maria Bogdan, Department of Pharmacology, University of Medicine and Pharmacy of Craiova, 2 Petru Rares Street, 200349 Craiova, Romania; bogdanfmaria81@yahoo.com; 6 Department of Anatomy, University of Medicine and Pharmacy of Craiova, 2 Petru Rares Street, 200349 Craiova, Romania; ancaenescus@yahoo.com; 7 Department of Medico-Surgical Emergencies, University of Medicine and Pharmacy of Craiova, 2 Petru Rares Street, 200349 Craiova, Romania; m_maria2521@yahoo.com (M.F.); sea180385@gmail.com (A.E.); 8 Faculty of Nursing and Midwifery, University of Medicine and Pharmacy of Craiova, 2 Petru Rares Street, 200349 Craiova, Romania; madeldum@yahoo.com; 9 Department of Medical Informatics and Biostatistics, University of Medicine and Pharmacy of Craiova, 2 Petru Rares Street, 200349 Craiova, Romania; dragosado@yahoo.com; 10 Department of Surgery, University of Medicine and Pharmacy of Craiova, 2 Petru Rares Street, 200349 Craiova, Romania; vsurlin@gmail.com; 11 Department of Pediatrics, University of Medicine and Pharmacy of Craiova, 2 Petru Rares Street, 200349 Craiova, Romania; petrescu.ileanaoctavia@yahoo.com; 12 Department of Plastic Surgery, University of Medicine and Pharmacy of Craiova, 2 Petru Rares Street, 200349 Craiova, Romania; meciurea@gmail.com; 13 Research Center of Gastroenterology and Hepatology Craiova, University of Medicine and Pharmacy of Craiova, 2 Petru Rares Street, 200349 Craiova, Romania; adrian.saftoiu@umfcv.ro or adriansaftoiu@aim.com; 14 Visiting Clinical Professor, Gastrointestinal Unit, Copenhagen University Hospital Herlev, 2730 Herlev, Denmark

**Keywords:** pancreatic disorders, polymorphism, genotype, VEGFR-2

## Abstract

Pancreatic disorders have a high prevalence worldwide. Despite the fact that screening methods became more effective and the knowledge we have nowadays about pancreatic diseases has enhanced, their incidence remains high. Our purpose was to determine whether single nucleotide polymorphism (SNP) of VEGFR-2/KDR (vascular endothelial growth factor receptor 2/kinase insert domain receptor) influences susceptibility to develop pancreatic pathology. Genomic DNA was extracted from blood samples collected from patients diagnosed with acute pancreatitis (*n* = 110), chronic pancreatitis (*n* = 25), pancreatic cancer (*n* = 82) and healthy controls (*n* = 232). VEGFR-2 (KDR) 604A>G (rs2071559) polymorphism frequency was determined with TaqMan allelic discrimination assays. Statistical assessment was performed by associating genetic polymorphism with clinical and pathological data. In both pancreatic disorders and healthy control groups the polymorphism we studied was in Hardy-Weinberg equilibrium. Association between increased risk for pancreatic disorders and studied polymorphism was statistically significant. KDR 604AG and AG + GG genotypes were more prevalent in acute pancreatitis and pancreatic cancer patients than in controls. These genotypes influence disease development in a low rate. No association was found between chronic pancreatitis and KDR 604AG and AG + GG genotypes. In Romanian cohort, we found an association between the KDR 604A→*G* polymorphism and acute pancreatitis and pancreatic cancer. Carriers of the -604G variant allele were more frequent among acute pancreatitis and pancreatic cancer than among controls, suggesting that KDR 604G allele may confer an increased risk for these diseases. In the future, more extensive studies on larger groups are necessary, in order to clarify the role of VEGFR2 polymorphisms in pancreatic pathology.

## 1. Introduction

Pancreatic ischemia, obstruction of the pancreatic bile duct, and activation of pancreatic protease and inflammatory cytokines represent the main causes reported in acute pancreatitis [1]. Little is known about the mechanisms responsible for disease onset and progression. The most frequent etiological factors are alcoholism and gallstones. Acute pancreatitis is characterized by an intense inflammatory response [1,2], a consequence of the imbalance between pro-inflammatory mediators and anti-inflammatory mechanisms [3,4]. In the pancreatic injury chain, several genetic factors, like cytokines (interleukins, IL-1, IL-1β, IL-1 receptor antagonist, IL-6, IL-10; tumor necrosis factor α, TNF-α) [2,5], angiogenesis-related factors (VEGFR-2; chemokine receptor 2, CXCR-2; proteinase-activated receptor 1, PAR-1; endothelial growth factor, EGF; tumoral growth factor β, TGF-β) [6,7,8,9] pattern-recognition receptor (CD14) [10] and inducible nitric oxide synthase (iNOS) [1] genes may play an important role, concerning severity, and evolution of the inflammatory or neoplastic processes.

Chronic pancreatitis is characterized histologically through dysplastic ducts, ductal cells proliferation, acinar cell degeneration, and fibrosis [7]. An increased risk to develop pancreatic cancer was described in patients with chronic pancreatitis [11]. Acute pancreatitis also seems to be the first step in this pathway of progression from chronic inflammation to pancreatic cancer [11]. Pancreatic cancer has the highest mortality among other types of cancer, and one of the worst prognosis among malignant solid tumours (survival rate is under 5%) [12]. Standard treatment in early-stage tumours implies surgical resection, thus encouraging improvement of early diagnosis methods that might lead to better results for these patients [12]. 

Thus, the progress of clinical and imaging diagnostic techniques is not enough to distinguish which patients have a poor prognosis and which have a better one. Recent studies show that molecular markers may serve as prognostic factors and can be used to identify aggressive pancreatic cancer phenotypes and choose the optimal individual therapies [7,8,12]. Promising prognostic markers may include genetic variations, like germline polymorphisms because they are stable, easily accessible, and unaffected by the genomic instability described in malignant tissue [6,13].

Angiogenesis is an important process for tumour progression, modulated by interactions between pro- and anti-angiogenic molecules. A main player in this process is represented by the VEGF pathway. VEGF contributes to the maintenance of mature functional vascularization and inhibition apoptosis of endothelial cells. Inhibition of VEGF disturbing the balance of apoptosis/proliferation of both endothelial cells and epithelial cells, which leads to an embryogenesis deficiency and severe lesions in the mature tissues [14]. VEGF family consists of several members: VEGF-A, PlGF (placental growth factor), VEGF-B, VEGF-C, VEGF-D, and their receptors [15]. The effects of VEGF are mediated by two receptor tyrosine kinase, VEGFR-1 (Flt-1, Fms-like tyrosine kinase receptor 1) and VEGFR-2 (KDR, Kinase insert domain receptor) [15]. KDR binds VEGF-A, VEGF-C and VEGF-D, this proteins playing a role in the regulation of expression of KDR [16]. Studies have shown that this receptor is considered to be the main messenger pathways for VEGF signaling in endothelial cells, which is induced chemotaxis and actin reorganization of endothelial proliferation [17,18,19].

The aim of the current study was to assess the role played by the VEGFR-2 (KDR) 604A>G (rs2071559) polymorphism in pancreatic pathology.

## 2. Results

We included in the study 449 patients, 217 with pancreatic disorders and 232 unaffected subjects. The mean age was 60.61 years (stdev.14.05) in the control group and 59.55 years (stdev.14.08) in studied cases. Thus, the two groups of subjects were similar, with homogenous demographic data according to age and sex ratio (Table 1). 

All 449 collected samples from patients with pancreatic disorders and healthy controls were genotyped. The polymorphism we studied was in Hardy-Weinberg equilibrium for both pancreatic disorders and healthy control groups.

**Figure 1:**
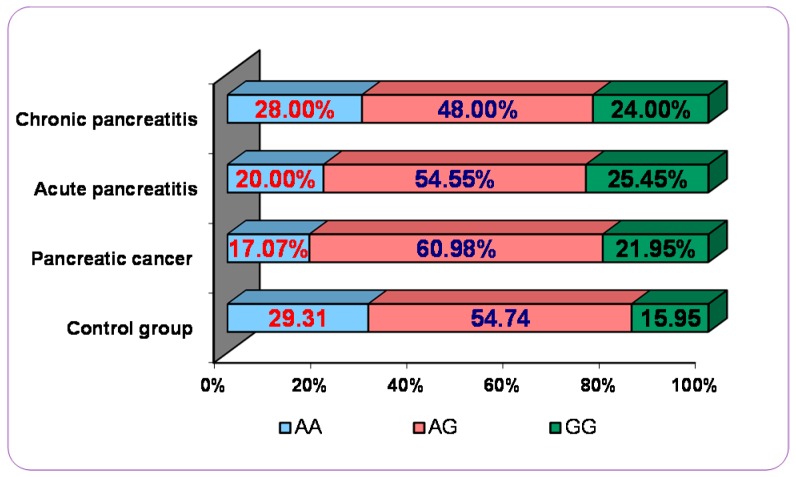
Polymorphism of VEGFR-2 (KDR) 604A>G in different pancreatic disorders vs. control group.

Genotype and allele frequencies of polymorphism KDR 604A>G (rs2071559) are listed for pancreatic disorders (acute pancreatitis, chronic pancreatitis, and pancreatic cancer) patients and control groups in Table 2 and Figure 1.

The comparative analysis of genotypes and statistical data obtained revealed that VEGFR-2 (KDR) 604A>G polymorphism had a strong association with pancreatic inflammatory pathology (acute pancreatitis).

As shown, we have found a statistically significant association between the presence of this polymorphism and the increased risk for patients to develop acute pancreatitis when we compared one genotype with other genotype (604 AA vs. GG:OR = 0.427, 95% CI: 0.215 to 0.850, *p* = 0.0154; or in a dominant model—G allele carriers, 604 AA vs. AG + GG:OR = 0.603, 95% CI: 0.349 to 1.041, *p* = 0.0494), (Table 2). In the study group variant GG (homozygous) of VEGFR-2 is available in more than 25% of patients, while in the control group, it was found in only 16% (Figure 1).

Additionally, we observed a statistically significant association between the presence of this polymorphism and the increased risk for patients to develop pancreatic cancer when we compared one genotype with another genotype (604 AA vs. AG:OR = 0.523, 95% CI: 0.270–1.014, *p* = 0.050; -604 AA vs. GG : OR = 0.423, 95% CI: 0.190 to 0.947, *p* = 0.036; or in a dominant model—G allele carriers, -604 AA vs. AG + GG: OR = 0.496, 95% CI: 0.262 to 0.943, *p* = 0.032) (Table 2). In the study group variant GG (homozygous) of VEGFR-2 is present in a lower percentage of 25 (21.95%), while in the control group was found in only 16% (Figure 1).

Results of our study showed that there are not any statistically significant association between VEGFR-2 (KDR) 604A>G polymorphism and the chronic pancreatitis: 604 AA vs. GG: OR = 0.635, 95% CI: 0.199 to 2.028, *p* = 0.4432; or in a dominant model—G allele carriers, 604 AA vs. AG + GG : OR = 0.938, 95% CI: 0.375 to 2.348, *p* =0.8911) (Table 2).

## 3. Discussion

Angiogenesis is an important process for the growth and development of all the tissues in which new blood vessels will take shape from preexisting vasculature. Disruption of the balance between pro- and anti-angiogenic molecules, can influence the process of angiogenesis [14,18,19].

Many studies have shown that VEGF and activation of the VEGF-receptor pathway in particular plays an important role in regulating angiogenic process. VEGF-receptor pathway promotes endothelial cell growth, survival, proliferation, migration, and differentiation, as well as vascular permeability and the mobilization of endothelial progenitor cells from the bone marrow [20,21,22,23,24]. Mukhopadhyay et al. showed in a study published in 2004 that in addition to its role in angiogenesis, VEGF is involved in the inflammatory process stimulating the synthesis of pro-inflammatory cytokines [25]. Also Kuehn et al. revealed by their research published in 1999 the strong expression of VEGF in ductal cells of chronic pancreatitis as well as in pancreatic cancer cells and showed for the first time that angiogenic activity is increased in both chronic pancreatitis and pancreatic adenocarcinoma [26].

In our study we aimed to investigate the involvement of VEGFR-2/KDR gene polymorphism (604A>G, rs2071559) in pancreatic pathology, in inflammation and neo-angiogenesis in pancreatic tumor. Following our analysis, as we presented the results we found a statistically significant association between this polymorphism and an increased risk for patients to develop acute pancreatitis and pancreatic cancer. Although we have achieved a statistically significant *p*, the value of OR suggests that this genotype influences the disease at a lower rate. We have not found similar studies in the specialty literature on which to relate.

Referring to other types of pancreatic diseases, we did not notice in our study a statistically significant association between this polymorphism and an increased risk for patients to develop chronic pancreatitis. We believe that the small number of patients diagnosed with this disease and included in the study may represent one of the reasons for the absence of statistically significant associations, which requires further studies and replication obtained data in cohorts that include a greater number of patients.

Angiogenesis-related factors were described to play an important role in the outcome of pancreatic cancer patients. In the last decade, several studies attempted to demonstrate the connection between polymorphisms of angiogenesis-related factors (VEGF, VEGFR-2, RET, EGF, TGF-β) genes and pancreatic tumours [6,8,9,27,28].

Studies also revealed that VEGF plays an important role in tumour genesis. Patients with colorectal cancer have a poorer overall survival which was associated with VEGFR-2 906 C/C genotype [29]. In non-small-cell lung cancer (NSCLC) Glubb et al. [30] prove a correlation between VEGFR-2 271A/A genotype and lower VEGFR-2 protein levels. In another recent study Kim et al. investigated the impact of five potentially functional polymorphisms in the VEGFA (rs699947, rs2010963, and rs3025039) and VEGFR2 (rs1870377 and rs2305948) genes on the survival of patients with diffuse large B cell lymphoma [20].

The impact of this polymorphism on VEGFR-2 mRNA and protein stability in pancreatic cancer is not known so far. Studies of VEGF gene polymorphisms (+405G/C but not −460T/C and +936C/T) demonstrated an association with susceptibility to pancreatic adenocarcinoma and this SNP has significant influence on serum VEGF level [21]. Recently, it has been shown that VEGFR2-906C>T polymorphism has a significant impact on the pancreatic cancer as a predictor for survival and tumour recurrence [6]. 

Thus, VEGFR2 polymorphisms may represent an important prognostic marker for pancreatic cancer [6]. Genotyping may, therefore, help to identify high-risk subgroups with potential benefit for patients suffering from pancreatic carcinoma [6]. In the present study, a statistical association between presence of the VEGFR2 (KDR) 604 A>G polymorphism and the risk of developing pancreatic pathology has been found. 

Another group of researchers investigated VEGF genotypes and serum concentration in patients with pancreatic adenocarcinoma (PA) and chronic pancreatitis (CP). They observed: an increased frequency of the homozygous +405C/C VEGF genotype in patients with PA compared with CP and control group, the distribution of genotype and allele frequencies of the −460C/T polymorphism in the PA patients did not differ from those in CP and control groups and serum levels of VEGF were significantly higher in PA patients compared with CP patients and control group. No relationship between VEGF serum levels and VEGF gene polymorphisms have been found. The authors concluded that +405C/C VEGF genotype may contribute to pancreatic carcinogenesis [31].

## 4. Material and Methods

### 4.1. Patients and Study Protocol

In this analysis of prospective collected data we included 217 subjects diagnosed with pancreatic disorders between October 2011 and November 2014 (chronic pancreatitis, acute pancreatitis, and pancreatic cancer), evaluated in the Gastroenterology Clinic, Medical II Clinic, and Surgery I Clinic, Emergency County Hospital, Craiova, Romania. In parallel, we investigated a control group that included 232 persons unaffected by pancreatic diseases.

The diagnosis of pancreatic cancer was established by imagistic methods, including EUS-guided FNA. In negative FNA patients the diagnosis was established after surgical resection completed with pathological exam or follow up at least six months in non-resectable tumours. Second or third EUS-guided FNA was also performed in a minority of patients.

The diagnosis of chronic pancreatitis was established by imagistic assessment: CT scan and EUS in patients with chronic alcohol consumption patients with early pancreatitis were excluded due to difficulties for certain diagnosis. The EUS criteria for chronic pancreatitis, according to the Rosemont classification, are listed in the Table 3. According to this consensus there are three types of results: consistent, suggestive and indeterminate chronic pancreatitis [32]. We included in the study only patients with consistent and suggestive chronic pancreatitis. 

The diagnosis of acute pancreatitis was established after CT scan with serum elevated pancreatic enzymes (lipase, amylase). For every case of acute pancreatitis we calculated the Ranson score, Balthazar score, and Atlanta classification, which divided a large number of cases of acute pancreatitis into mild, moderate, and severe subgroups.

Inclusion and exclusion criteria for this study are represented in the Table 4.

All cases and controls were of Romanian ethnic origin and consented to the study. Demographic data, age, gender, body mass index, diabetes, and clinical information were registered for each patient.

The study design was approved by the Ethics Committee of University of Medicine and Pharmacy of Craiova, Romania. 

### 4.2. SNP Genotyping

Genomic DNA was purified from peripheral blood leukocytes collected from all the subjects included in this study (110: acute pancreatitis, 25: chronic pancreatitis, 82: pancreatic cancer, 232: controls), using a Wizard^®^ Genomic DNA Purification Kit (Promega, Madison, WI, USA). All participants were genotyped for VEGFR-2 (KDR) 604A>G (rs2071559) in the Human Genomic Laboratory, University of Medicine and Pharmacy of Craiova. The genotyping was performed with TaqMan probes following the protocol recommended by the supplier (Applied Biosystems, Foster City, CA, USA). The allelic discrimination option of ViiA™ 7 Software v1.0 was used to interprete the results. 

### 4.3. Statistical Analysis

The Hardy-Weinberg equilibrium was tested to compare the observed and expected genotype frequencies among cases and controls. To estimate the association between VEGFR-2 (KDR) polymorphism and pancreatic pathology, we calculated odds ratios (ORs) and 95% confidence intervals (95% CI) using logistic regression analysis. Genotypes were assessed using indicator variables with the common homozygote as reference. An analysis of contingency table was performed for categorical data with two-tailed *p*-value was obtained using Fisher's exact test while unpaired *t*-test was used for continuous data. A two-sided *p*-value < 0.05 was considered to be statistically significant.

## 5. Conclusions

In the Romanian cohort, we found an association between the KDR 604A→G polymorphism and acute pancreatitis and pancreatic cancer. Carriers of the 604G variant allele (single genotypes, combined genotypes) were more frequent among acute pancreatitis and pancreatic cancer than among controls, suggesting that the KDR 604G allele may confer an increased risk for these diseases. In the future, more extensive studies on larger groups are necessary, in order to clarify the role of VEGFR2 polymorphisms in pancreatic pathology.

## Figures and Tables

**Table 1:** Demographic characteristics of the patients

Characteristic	Pancreatic Disorders *n* = 217	Control Subjects *n* = 232	*p*-Value
Age (year)	59.55 (stdev.14.08)	60.61 (stdev.14.05)	0.496 (unpaired *t*-test)
Sex: male/female	113/104	122/110	0.924 (Fisher’s exact test)

**Table 2:** Polymorphism of VEGFR-2 (KDR) 604A>G in different pancreatic disorders.

**VEGFR-2 (KDR) 604A>G**	**Acute Pancreatitis**	**Control**	**OR (95% CI)**	***p***
AA	22 (20.00%)	68 (29.31%)	reference	
AG	60 (54.55%)	127 (54.74%)	0.685 (0.387 to 1.211)	0.1933
GG	28 (25.45%)	37 (15.95%)	0.427 (0.215 to 0.850)	0.0154 *
G allele carriers	88 (80.00%)	164 (70.69%)	0.603 (0.349 to 1.041)	0.0494 *
**VEGFR-2 (KDR) 604A>G**	**Chronic Pancreatitis**	**Control**	**OR (95% CI)**	***p***
AA	7 (28.00%)	68 (29.31%)	reference	
AG	12 (48.00%)	127 (54.74%)	1.089 (0.410 to 2.896)	0.8636
GG	6 (24.00%)	37 (15.95%)	0.635 (0.199 to 2.028)	0.4432
G allele carriers	18 (72.00%)	164 (70.69%)	0.938 (0.375 to 2.348)	0.8911
**VEGFR-2 (KDR) 604A>G**	**Pancreatic Cancer**	**Control**	**OR (95% CI)**	***p***
AA	14 (17.07%)	68 (29.31%)	reference	
AG	50 (60.98%)	127 (54.74%)	0.523 (0.270 to 1.014)	0.050 *
GG	18 (21.95%)	37 (15.95%)	0.423 (0.190 to 0.947)	0.036 *
G allele carriers	68 (82.93%)	164 (70.69%)	0.496 (0.262 to 0.943)	0.032 *

* *p* statistically significant.

**Table 3:** Diagnostic EUS criteria for chronic pancreatitis [32].

Major A Criteria	Major B Criteria	Minor Criteria
Hyperechoic foci with shadowing, main pancreatic duct (MPD) calculi	Lobularity, honeycombing type	Cysts, Dilated MPD (≥3.5 mm), irregular MPD contour, Dilated side branches (≥1 mm), hyperechoic duct wall, hyperechoic non-shadowing foci, non-honeycombing lobularity

**Table 4:** Inclusion and exclusion criteria for the study.

Patients with Pancreatic Disorders		Healthy Subjects	
Inclusion criteria	Exclusion criteria	Inclusion criteria	Exclusion criteria
Age 18–90 years	Age <18 years	Age 18–90 years	Age <18 years
Certain diagnosis of chronic pancreatitis, acute pancreatitis or pancreatic cancer	Absence of pancreatic disorders	Absence of pancreatic disorders	Other cancers
Signed informed consent	Pancreatic neuroendocrine tumours Other cancers	Signed informed consent	
